# Association between postoperative bowel symptoms and social isolation in patients with rectal cancer: the moderating effects of social support and psychological resilience

**DOI:** 10.3389/fpubh.2026.1963156

**Published:** 2026-10-14

**Authors:** Yaolan Wang, Lin Yao, Xinlei Zhao, Xiaodong Wang, Mingjun Huang

**Affiliations:** 1 West China School of Nursing/Day Surgery Center, West China Hospital, Sichuan University, Chengdu, China; 2 Division of Gastrointestinal Surgery, Department of General Surgery, West China Hospital, West China School of Medicine, Sichuan University, Chengdu, China; 3 West China School of Nursing/Division of Gastrointestinal Surgery, Department of General Surgery, West China Hospital, Sichuan University, Chengdu, China; 4 Division of Gastrointestinal Surgery, Department of General Surgery, West China Hospital, Sichuan University, Chengdu, China; 5 Day Surgery Center / West China School of Nursing, West China Hospital, Sichuan University, Chengdu, China

**Keywords:** bowel symptoms, moderating effect, perceived social support, psychological resilience, rectal cancer, social isolation, sphincter-preserving surgery

## Abstract

**Background:**

60% ~ 90% of patients with rectal cancer undergoing sphincter-preserving surgery experience persistent bowel symptoms, and patients also report varying degrees of social isolation. Those bowel symptoms are often particularly pronounced during the first month after surgery, when patients may also experience substantial challenges in resuming social participation. However, evidence regarding the association between bowel symptoms and social isolation remains limited, and the potential moderating roles of psychosocial resources are not well understood.

**Objective:**

To investigate the association between postoperative bowel symptoms and social isolation among patients with rectal cancer following sphincter-preserving surgery, and to further examine the moderating effects of perceived social support and psychological resilience.

**Methods:**

This secondary analysis used data from an ongoing prospective observational cohort study of patients with rectal cancer undergoing sphincter-preserving surgery at a tertiary hospital in China. Demographic and clinical characteristics, perceived social support, and psychological resilience were assessed preoperatively, while bowel symptoms and social isolation were assessed at the 1-month postoperative follow-up. Baseline and 1-month postoperative data were used in this study. Spearman correlation analysis and hierarchical multiple linear regression analysis were performed to examine the association between postoperative bowel symptoms and social isolation. Simple slope analysis and Johnson–Neyman (J-N) analysis were conducted to evaluate the moderating roles of perceived social support and psychological resilience.

**Results:**

A total of 351 patients were included in the final analysis. Postoperative bowel symptoms were significantly and positively correlated with social isolation (*r_s_* = 0.595, *p* < 0.001). After adjusting for relevant demographic and clinical factors, bowel symptoms remained independently associated with higher levels of social isolation (B = 0.506, *p* < 0.001). Separate moderation analyses showed significant interactions for perceived social support (B = −0.020, 95% CI −0.032 to −0.009, ΔR^2^ = 0.018, *p* = 0.001) and psychological resilience (B = −0.011, 95% CI −0.019 to −0.004, ΔR^2^ = 0.013, *p* = 0.003), although the additional variance explained by the interaction terms was modest. Simple slope analyses indicated that the association between bowel symptoms and social isolation remained significant across low, mean, and high levels of both moderators. Johnson–Neyman analysis showed that the association became nonsignificant only within a narrow range at the upper end of the observed social support distribution (actual scores ≥ approximately 69), whereas it remained significant across the entire observed range of psychological resilience.

**Conclusion:**

Postoperative bowel symptom burden was positively associated with social isolation among patients with rectal cancer following sphincter-preserving surgery. Higher perceived social support and psychological resilience were associated with weaker bowel symptom–social isolation relationships. These findings support an integrated supportive-care approach that combines bowel symptom management with psychosocial and social support to address postoperative social isolation and facilitate social functioning.

## 1 Introduction

Colorectal cancer (CRC) is one of the most common malignancies of the digestive system. According to the latest GLOBOCAN 2024 estimates released by the International Agency for Research on Cancer (IARC), CRC ranks third in incidence and second in mortality among all cancers worldwide, with more than 2.0 million new cases annually, of which rectal cancer accounts for 37.9% (1). Surgery remains the primary curative treatment for rectal cancer. In this context, sphincter-preserving surgery has become one of the main surgical approaches for patients with rectal cancer, as it avoids permanent stoma formation and preserves anal sphincter function (2). However, approximately 60–90% of patients undergoing sphincter-preserving surgery experience bowel symptoms, such as increased stool frequency, urgency, and even fecal incontinence (3). These bowel symptoms are typically more severe in the early postoperative period, especially during the first month after surgery, and gradually improve over time, reaching a relatively stable state at approximately 12 months after surgery (4). Nevertheless, approximately 47.5–90% of patients continue to experience persistent bowel symptoms 4–15 years after surgery (5, 6), which significantly impairs their long-term quality of life (7).

Beyond the physiological burden, persistent bowel symptoms are associated with impaired social functioning and disruptions in daily life (8). Because of concerns about sudden defecation and fecal incontinence, patients often experience behavioral changes such as restricted outings, reduced social participation, and social withdrawal. This is most pronounced during the first month following surgery, as patients need to adapt to the lifestyle changes brought about by their bowel symptoms (9). These changes have been reported to be associated with psychological distress, including anxiety, depressive symptoms, and increased feelings of social disconnection, which are closely related to social isolation among patients after surgery (10). Social isolation is characterized as an automatic process of social withdrawal involving both psychological and behavioral dimensions. The subjective dimension refers to individuals’ perceived feelings of loneliness and helplessness, whereas the objective dimension reflects observable changes in social participation with the external environment, such as social withdrawal, reduced frequency and scope of social interactions, and impaired social functioning (11). Accordingly, social isolation in the present study was conceptualized as a multidimensional construct encompassing both subjective emotional disconnection and behavioral disengagement from social interaction. The former is reflected by emotional isolation, whereas the latter is reflected by reduced interpersonal interaction, social withdrawal, and restricted social participation. Accumulating evidence suggests that social isolation is closely associated with multiple adverse outcomes in cancer patients. In terms of mental health, social isolation has been significantly linked to higher levels of depression and anxiety (12, 13). Regarding social functioning, it is associated with impaired role functioning and reduced social participation, making it difficult for patients to readjust to family and occupational roles (14). Moreover, social isolation has been identified as an independent risk factor for increased all-cause mortality among cancer patients (15). Therefore, a deeper understanding of the intrinsic relationship between bowel symptoms and social isolation is essential for improving psychosocial adaptation and overall quality of life in patients following rectal cancer surgery.

Currently, multiple qualitative studies based on patient interviews have highlighted the relationship between bowel symptoms and social isolation. A systematic review indicates that patients following sphincter-preserving surgery for rectal cancer often “live in uncertainty” due to unpredictable bowel urgency and fecal incontinence, and gradually develop social withdrawal, reduced social participation, and an increased sense of social disconnection. Patients frequently restrict going out and participating in social activities due to concerns about embarrassment and negative evaluation of their symptoms, thereby progressively entering a state of social isolation (9, 16). These qualitative findings suggest that bowel symptoms may be an important factor associated with social isolation. However, several limitations remain in the current literature regarding the association between bowel symptoms and social isolation. First, few studies have directly examined their quantitative relationship; most existing research has only indirectly described the association between bowel symptoms and social functioning or psychological distress (17), lacking systematic analyses with social isolation as an explicit outcome. Second, potential moderating or buffering factors have not been sufficiently explored, and it remains unclear why individuals with similar levels of bowel symptoms may experience different degrees of social functional impairment (18, 19). Therefore, identifying psychological and social resources that may attenuate the association between bowel symptoms and social isolation is important for understanding individual differences in psychosocial adaptation and informing future intervention studies.

Social support and psychological resilience, as key psychosocial resources representing external and internal domains respectively, may play a significant moderating role in the relationship between bowel symptoms and social isolation (19, 20). According to the stress-buffering theory, both external social resources (e.g., social support) and internal psychological resources (e.g., psychological resilience) can exert protective effects when individuals are exposed to stressors (21, 22). Specifically, social support provides emotional, informational, and instrumental assistance that can effectively reduce the adverse impact of stressful events on individuals’ psychological and physical adaptation. In the context of rectal cancer surgery, a higher level of social support may alleviate patients’ feelings of shame and anxiety associated with the unpredictability of bowel symptoms, which may facilitate social participation and attenuate the association between bowel symptoms and social isolation (23). Psychological resilience refers to an individual’s capacity to adapt and recover in the face of adversity and stress. Patients with higher levels of psychological resilience are more likely to employ adaptive coping strategies, effectively coping with psychological burden and functional limitations associated with bowel symptoms, which may be related to less social withdrawal and better maintenance of social participation (24). Taken together, social support and psychological resilience constitute an important protective system for coping with disease-related stress from external and internal perspectives, respectively. The former enhances access to external social resources, whereas the latter strengthens individuals’ internal adaptive capacity. Both may therefore exert moderating effects on the relationship between bowel symptoms and social isolation.

Based on the above, this study aims to: (1) examine the association between bowel symptoms and social isolation in patients following sphincter-preserving surgery for rectal cancer; and (2) investigate the moderating effects of social support and psychological resilience on the relationship between bowel symptoms and social isolation. Accordingly, the following hypotheses are proposed (see Figure 1):

**Figure 1:**
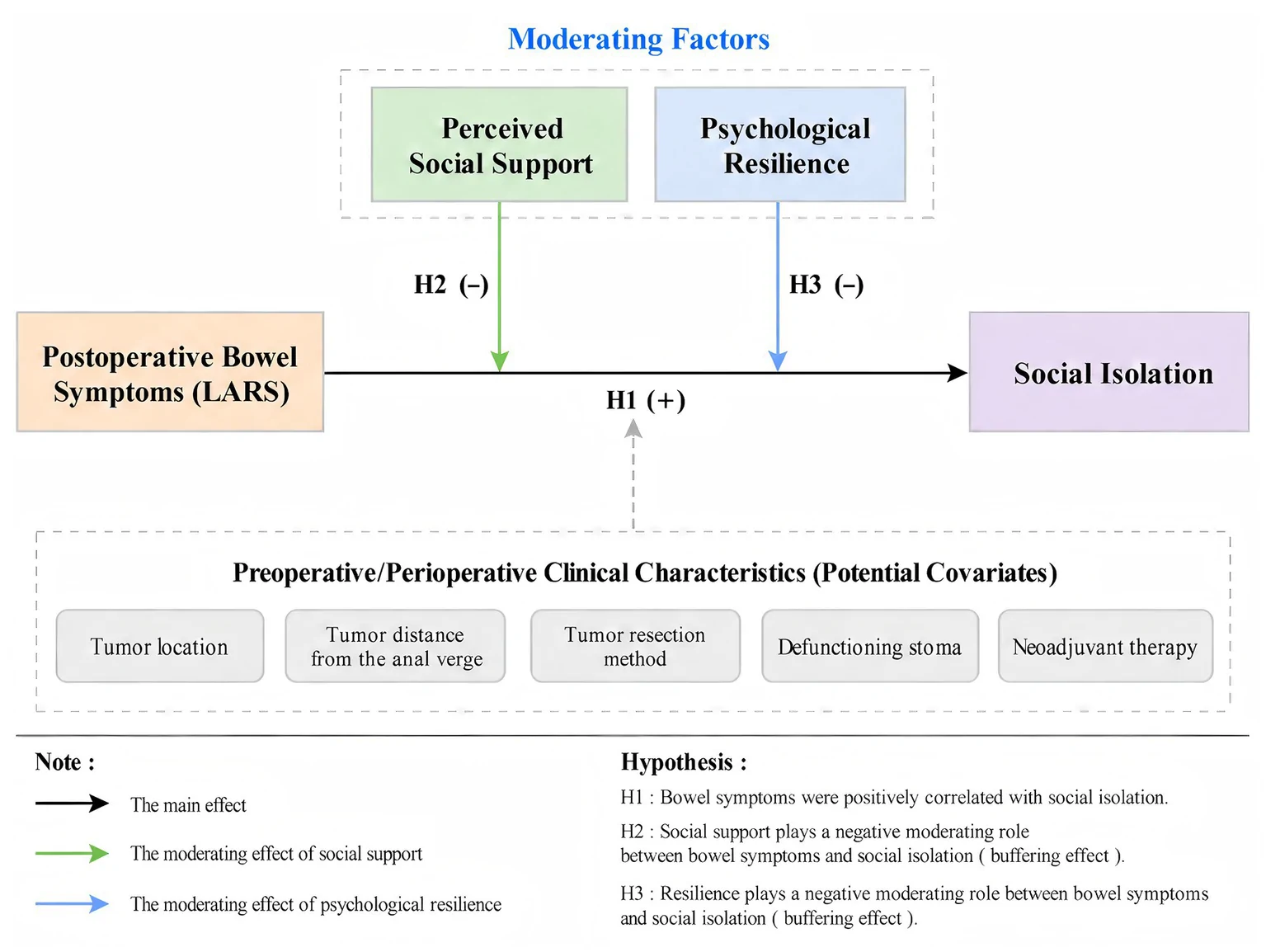
Conceptual framework for moderation analysis of the association between postoperative bowel symptoms and social isolation.

*H1*: Bowel symptoms are positively associated with social isolation.

*H2*: Social support moderates the relationship between bowel symptoms and social isolation, such that the positive association is weaker among patients with higher levels of social support.

*H3*: Psychological resilience moderates the relationship between bowel symptoms and social isolation, such that the positive association is weaker among patients with higher levels of psychological resilience.

By examining the association between bowel symptoms and social isolation in patients following sphincter-preserving surgery for rectal cancer, and further exploring the moderating roles of social support and psychological resilience, this study may provide a better understanding of how postoperative bowel symptoms are related to social isolation and why individuals with similar symptom burdens may experience different levels of social isolation. The findings may provide insights into potential psychosocial resources that could be considered in supportive care strategies for rectal cancer survivors.

## 2 Method

### 2.1 Study design

This study was a secondary analysis of data from an ongoing prospective observational cohort study being conducted from June 2025 to October 2026 at a tertiary hospital in China. The parent cohort was designed to investigate the longitudinal trajectories of bowel symptoms and social isolation among patients with rectal cancer undergoing sphincter-preserving surgery. At baseline (before surgery), demographic characteristics, disease- and treatment-related information, perceived social support, and psychological resilience were collected. Patients were subsequently followed up at 1, 3, and 6 months after surgery to assess bowel symptoms and social isolation.

For the present study, baseline data and 1-month postoperative follow-up data (The main reason is that bowel symptoms are typically most severe during the early postoperative period; therefore, the 1-month assessment captures a clinically relevant phase characterized by substantial symptom burden and pronounced challenges in resuming social participation) were extracted from the cohort database to examine the association between postoperative bowel symptoms and social isolation, and to further explore the moderating roles of perceived social support and psychological resilience on this association. This study was reported in accordance with the Strengthening the Reporting of Observational Studies in Epidemiology (STROBE) guidelines.

### 2.2 Participants

Participants were recruited from the parent cohort and included patients with rectal cancer who underwent sphincter-preserving surgery. Eligible participants met the following inclusion criteria: (1) aged 18 years or older; (2) pathologically confirmed diagnosis of rectal cancer; (3) underwent sphincter-preserving radical surgery, with or without a temporary defunctioning stoma; (4) provided written informed consent before participation, and (5) patients who completed the 1-month follow-up assessment were included in the final analysis. Patients were excluded if they: (1) had a history of other malignant tumors; (2) underwent permanent colostomy; (3) had pre-existing gastrointestinal disorders that could affect bowel function assessment (e.g., inflammatory bowel disease or irritable bowel syndrome); or (4) had severe cognitive impairment, psychiatric disorders, or communication difficulties that prevented questionnaire completion.

A total of 408 patients were enrolled at baseline. One month after surgery, data were collected through structured telephone follow-up. Of these, 351 patients completed the 1-month postoperative assessment, resulting in a follow-up completion rate of 86.0% and a loss-to-follow-up rate of 14.0%.

### 2.3 Data collection

At admission, the researchers briefly introduced the study objectives and procedures of the study in a quiet and private setting. Participants who agreed to participate were asked to sign an informed consent form and then complete questionnaires assessing sociodemographic characteristics, psychological resilience, and perceived social support. All questionnaires were administered in a quiet inpatient setting, with standardized written and verbal instructions provided to ensure independent completion. Research staff were available to clarify procedural issues only and were strictly instructed not to influence participant responses. During hospitalization, clinical and treatment-related variables were extracted from the hospital electronic medical record system by trained investigators; Regarding the follow-up assessments, patients without a stoma were assessed one month after sphincter-preserving surgery, whereas patients with a temporary defunctioning stoma were assessed one month after stoma reversal. Importantly, all follow-up assessments were conducted after restoration of intestinal continuity and resumption of fecal passage through the anus. At the 1-month postoperative follow-up, outcome data were collected through structured telephone interviews. The severity of bowel symptoms and levels of social isolation were assessed at this stage. To ensure standardization, all interviewers underwent formal training and followed a structured interview guide. During each interview, items were read verbatim from the validated scales, and responses were recorded without interpretation, prompting, or modification. To ensure data quality, all completed records were independently checked by two investigators for completeness and logical consistency. Any discrepancies or missing information were resolved through re-examination of the original records or, when necessary, follow-up contact with participants.

### 2.4 Measures

#### 2.4.1 Sociodemographic and clinical variables

A structured questionnaire developed based on previous literature and clinical practice was used to collect participants’ sociodemographic and clinical characteristics. Sociodemographic variables included age, sex, body mass index (BMI), education level, residence type (urban/rural), living status, marital status, employment status, and primary family caregiver. Clinical and treatment-related information was obtained to capture disease and perioperative status, including tumor TNM stage, tumor location, tumor distance from the anal verge, receipt of neoadjuvant therapy, tumor resection method, presence of a defunctioning stoma, adjuvant therapy, postoperative complications, and length of hospital stay.

#### 2.4.2 Low anterior resection syndrome score

The Low Anterior Resection Syndrome (LARS) scale was developed by Emmertsen and colleagues in Denmark (25) based on a national colorectal cancer database. This tool is primarily used to assess the severity of bowel symptoms after sphincter-preserving surgery for rectal cancer. It consists of five core items assessing flatus incontinence, liquid stool incontinence, stool frequency, clustering of bowel movements, and urgency. The total score ranges from 0 to 42, with patients classified into three categories according to severity: no bowel symptoms (0–20), minor bowel symptoms (21–29), and major bowel symptoms (30–42). In the present study, the total LARS score was analyzed as a continuous variable in regression analyses. The Chinese version of the scale was translated and culturally adapted by Cao et al. (26) in 2013. It demonstrated excellent reliability, with an intraclass correlation coefficient (ICC) of 0.9615 for test–retest reliability and 0.9394 for inter-rater reliability, as well as good content validity (content validity ratio, CVR = 0.90). These findings indicate that the adapted scale has strong psychometric properties. Furthermore, Yan et al. (27) applied the Chinese version of the LARS scale in 108 patients after low anterior resection and reported a sensitivity of 93.8% and specificity of 76.7%, confirming its applicability and validity in Chinese colorectal cancer populations.

#### 2.4.3 Social isolation scale for colorectal cancer survivors

Social isolation was assessed using the Social Isolation Scale for Colorectal Cancer Survivors developed by Wang et al. (28). The scale was constructed based on a theoretical model of social isolation and includes 16 items across two dimensions: social disengagement and emotional isolation. The social disengagement dimension primarily reflects behavioral manifestations of social isolation, such as social withdrawal and reduced social participation, whereas the emotional isolation dimension primarily reflects subjective experiences of emotional disconnection. Thus, these two dimensions broadly correspond to the objective and subjective aspects of social isolation described in the conceptual framework, respectively. Each item is rated on a 5-point Likert scale ranging from 1 (strongly disagree) to 5 (strongly agree), with total scores ranging from 16 to 80. Higher scores indicate a higher level of social isolation. The scale has shown strong psychometric properties, with Cronbach’s α coefficients of 0.923 for the total scale, 0.853 for the social disengagement dimension, and 0.905 for the emotional isolation dimension. Its application in Chinese colorectal cancer populations further confirmed excellent reliability, with a Cronbach’s α of 0.958 (29).

#### 2.4.4 Perceived Social Support Scale (PSSS)

Perceived social support was assessed using the Perceived Social Support Scale (PSSS), originally developed by Zimet et al. (30) and validated in Chinese populations by Jiang et al. (31). The scale comprises 12 items across three domains: family support, friend support, and support from significant others. Each item is rated on a 7-point Likert scale ranging from 1 (very strongly disagree) to 7 (very strongly agree), yielding a total score between 12 and 84. Higher scores indicate a greater level of perceived social support. The Chinese version of the PSSS has demonstrated good reliability and validity, with a Cronbach’s α of 0.887.

#### 2.4.5 Connor–Davidson Resilience Scale

Psychological resilience was measured using the Connor–Davidson Resilience Scale (CD-RISC), developed by Connor and Davidson (32) and adapted into Chinese by Yu et al. (33). The scale consists of 25 items covering three dimensions: tenacity, strength, and optimism. Each item is rated on a 5-point Likert scale from 0 (not true at all) to 4 (true nearly all the time), with total scores ranging from 0 to 100. Higher scores reflect greater resilience, which can be categorized as low (0–40), moderate (41–80), and high (81–100). In the present study, resilience scores were treated as a continuous variable in statistical analyses. The Chinese version has demonstrated excellent internal consistency, with a Cronbach’s α of 0.913.

### 2.5 Statistical analysis

All statistical analyses were performed using SPSS Statistics version 27.0 and R software version 4.3.1. A two-sided *p* value <0.05 was considered statistically significant.

#### 2.5.1 Processing of missing data

In accordance with data handling guidelines, variables with >20% missing data were excluded. For variables with <20% missing data, multiple imputation by chained equations (MICE) was performed using SPSS version 27.0, assuming a missing-at-random (MAR) mechanism. Ten imputed datasets were generated, and estimates were pooled according to Rubin’s rules. A complete-case sensitivity analysis was additionally performed to assess the robustness of the findings to the missing-data handling approach.

#### 2.5.2 Descriptive statistics

Descriptive analyses were conducted to summarize participants’ sociodemographic characteristics, clinical variables, and scale scores. Normally distributed continuous variables were expressed as mean ± standard deviation (SD), while non-normally distributed variables were presented as median and interquartile range (IQR). Categorical variables were summarized as frequencies and percentages.

#### 2.5.3 Univariate analyses

Univariate analyses were conducted to identify potential sociodemographic and clinical variables associated with social isolation. For categorical variables, differences in social isolation scores between groups were compared using the independent-samples *t*-test for dichotomous variables or one-way analysis of variance (ANOVA) for variables with three or more categories, otherwise, the Mann–Whitney U test or Kruskal–Wallis H test was used, as appropriate. For continuous variables, associations with social isolation were examined using Pearson correlation analysis for normally distributed variables or Spearman rank correlation analysis for non-normally distributed variables. Candidate covariates were selected based on previous literature, clinical relevance, and their potential relationships with postoperative bowel symptoms and social isolation. Variables showing associations with social isolation at *p* < 0.10 in univariate analyses were entered into the hierarchical regression models.

#### 2.5.4 Hierarchical multiple regression analyses

Two separate hierarchical multiple linear regression models were constructed to examine whether perceived social support and psychological resilience moderated the association between postoperative bowel symptoms and social isolation, respectively. All continuous variables were mean-centered prior to analysis. Variance inflation factors (VIFs) were calculated to assess multicollinearity, with VIF values < 10 indicating acceptable levels. The assumptions of multiple linear regression were assessed for the final moderation models. Linearity and homoscedasticity were evaluated by visual inspection of scatterplots of standardized residuals against standardized predicted values. The normality of residuals was assessed using residual histograms and normal P–P plots. Potentially influential observations were examined using studentized residuals, leverage values, and Cook’s distance.

In Step 1, sociodemographic and clinical variables that showed significance in univariate analyses (*p* < 0.10) were entered as covariates. In Step 2, postoperative bowel symptoms (LARS score) were entered to examine their association with social isolation after adjustment for covariates. In Step 3, perceived social support or psychological resilience were included to examine their independent associations with social isolation after controlling for covariates and postoperative bowel symptoms. In Step 4, interaction terms (bowel symptoms × social support or bowel symptoms × psychological resilience) were entered to examine whether social support and psychological resilience moderated the association between bowel symptoms and social isolation (34).

For each significant interaction effect, simple slope analyses were conducted to examine the association between bowel symptoms and social isolation at low (−1 SD), mean, and high (+1 SD) levels of the moderators. Simple slopes and 95% confidence intervals were estimated to determine statistical significance. Interaction effects were visualized using the “ggplot2” package in R software. In addition, the Johnson–Neyman technique was applied to identify regions of significance, indicating the range of moderator values at which the association between bowel symptoms and social isolation was statistically significant (35).

### 2.6 Quality control

Several measures were implemented to ensure the quality and reliability of data collection and analysis. Before study initiation, all research staff involved in participant recruitment, questionnaire administration, and telephone follow-up received standardized training on study procedures, questionnaire administration protocols, and communication techniques.

During baseline assessment, participants completed questionnaires independently under standardized instructions in a quiet and private environment. Research staff were available only to clarify procedural issues and avoided influencing participants’ responses. For the 1-month postoperative follow-up, trained interviewers conducted structured telephone interviews using a standardized interview guide. All scale items were read verbatim, and responses were recorded without interpretation, prompting, or modification to minimize interviewer bias. Completed questionnaires and interview records were reviewed independently by two investigators to verify completeness, accuracy, and logical consistency. Any discrepancies or missing information were resolved by reviewing the original data sources or contacting participants when necessary.

Regarding data management and statistical analysis, data were systematically checked for completeness, accuracy, and outliers before analysis. Statistical analyses followed a predefined analysis plan. Continuous variables were examined for distributional assumptions before selecting appropriate statistical tests. Multicollinearity among predictors was assessed using variance inflation factors (VIFs), and interaction effects were further evaluated using simple slope analyses and Johnson–Neyman techniques to ensure robust interpretation of moderation effects.

### 2.7 Ethical considerations

This study was approved by the Ethics Committee of West China Hospital, Sichuan University (Approval No. 2025(1538)). All participants provided written informed consent before participation. The study was conducted in accordance with the Declaration of Helsinki and relevant ethical guidelines for human research.

## 3 Results

A total of 408 patients with rectal cancer who underwent sphincter-preserving surgery were initially enrolled in this cohort study. At the 1-month postoperative follow-up, 351 patients completed the assessment and 57 were lost to follow-up, corresponding to a loss-to-follow-up rate of 14.0%. Finally, 351 patients were included in the final analysis. To assess the potential selection bias attributable to loss to follow-up, we compared the baseline characteristics of participants who completed the follow-up (*n* = 351) with those who did not complete the follow-up (*n* = 57), based on the observed baseline data. The results showed that the two groups were comparable with respect to baseline characteristics (see Supplementary Table S1).

Among the 351 participants included in the present analysis, four variables (TNM staging: 9.4%, adjuvant treatment: 2.3%, tumor distance from the anal verge: 0.9%, and postoperative hospital stay: 0.3%) had missing values (see Supplementary Table S2), with missing rates ranging from 0.3 to 9.4%. Multiple imputation was performed under the assumption that the data were missing at random (MAR), and 10 imputed datasets were generated. Subsequent analyses were conducted using the pooled estimates from the 10 imputed datasets according to Rubin’s rules. In addition, a complete-case sensitivity analysis was conducted (*n* = 310), and the results were broadly consistent with those of the primary analysis (see Supplementary Tables S5–S7).

### 3.1 Participants characteristics and descriptive statistics

Regarding demographic characteristics, the majority of participants were male (*n* = 214, 60.97%), with a median age of 61 years (IQR: 55–69). The distribution of body mass index (BMI) categories showed that most participants were classified as having a normal BMI (*n* = 195, 55.56%) or being overweight (*n* = 115, 32.76%). Most participants had an educational level of elementary school or below (*n* = 118, 33.62%) and middle school education (*n* = 168, 47.86%) accounting for more than 80% of the sample. Most participants lived in urban areas (*n* = 223, 63.53%), lived with their spouse (*n* = 295, 84.05%), and were married (*n* = 304, 86.61%). Regarding working status, the proportions of employed participants (*n* = 156, 44.44%) and those who had resigned or retired (*n* = 177, 50.43%) were comparable. The spouse was the most common primary family caregiver (*n* = 218, 62.11%).

Regarding clinical and tumor-related characteristics, tumor location was predominantly ultra-low rectal position (*n* = 124, 35.33%) and low rectal position (*n* = 90, 25.64%), together accounting for more than 60% of cases. The median tumor distance from the anal verge was 5 cm (IQR: 3–8). Regarding TNM staging, stage III disease was the most common (*n* = 209, 59.54%). The most frequently performed tumor resection method was ultra-low anterior resection (*n* = 148, 42.17%). A total of 205 patients (58.40%) underwent defunctioning stoma. Neoadjuvant treatment was administered to 164 patients (46.72%), whereas 217 patients (61.82%) received adjuvant treatment after surgery. Postoperative complications were reported in 33 patients (9.40%). The median postoperative hospitalization duration was 8 days (IQR: 7–10).

For the main psychosocial and symptom-related variables, the median scores of psychological resilience and perceived social support were 55 (IQR: 47–64) and 54 (IQR: 50–59), respectively. The median social isolation score was 55 (IQR: 46–61). The median LARS score was 29 (IQR: 22–34). The Participant characteristics and descriptive statistics are shown in Tables 1, 2.

**Table 1:** Demographic and clinical characteristics of participants and their associations with social isolation.

Variable	Total (*n* = 351)	Social isolation (IQR)	Statistics	*p*
Gender (*n*, %)				
Male	214 (60.97%)	54 (46–61)	U = 13,438	0.188
Female	137 (39.03%)	56 (48–61.5)		
BMI (*n*, %)				
Slender	21 (5.98%)	50 (42.5–59)	H = 2.436	0.487
Normal	195 (55.56%)	55 (48–59)		
Overweight	115 (32.76%)	55 (46–65)		
Obesity	20 (5.70%)	57.5 (43.5–67.75)		
Level of education (*n*, %)				
Elementary school and below	118 (33.62%)	56 (48–62)	H = 5.005	0.171
Middle school	168 (47.86%)	55 (47.25–61.75)		
Junior college	40 (11.40%)	52 (45.25–60.25)		
Bachelor’s degree and above	25 (7.12%)	49 (40–58)		
Residence type (*n*, %)				
Rural	128 (36.47%)	55 (48–63.5)	U = 12,826	0.114
Towns	223 (63.53%)	54 (46–60)		
Living status (*n*, %)				
Living alone	30 (8.55%)	57 (47.5–65)	H = 2.910	0.233
Living with spouse	295 (84.05%)	55 (46–61)		
Living with others	26 (7.41%)	49 (46–59.25)		
Marital status (*n*, %)				
Married	304 (86.61%)	54 (46–61)	H = 0.540	0.763
Unmarried	5 (1.42%)	57 (41.5–63)		
Divorce or widowhood	42 (11.97%)	56 (46–62.5)		
Working status (*n*, %)				
Employed	156 (44.44%)	54 (48–61)	H = 0.912	0.634
Resignation or retirement	177 (50.43%)	55 (46–60.5)		
Unemployed	18 (5.13%)	56.5 (47.5–65)		
Primary family caregiver (*n*, %)				
No caregiver	25 (7.12%)	56 (47.5–65)	H = 2.259	0.323
Spouse	218 (62.11%)	53 (46–61)		
Other caregivers	108 (30.77%)	55.5 (47–61)		
Tumor location (*n*, %)				
Ultra-low rectal position	124 (35.33%)	59 (54–67)	H = 86.676	<0.001
Low rectal position	90 (25.64%)	56 (50.75–61.25)		
Medium rectal position	84 (23.93%)	50 (43.25–55)		
High rectal position	53 (15.10%)	42 (37–53)		
TNM staging (*n*, %)				
I Stage	37 (10.54%)	52 (46–67)	H = 2.065	0.559
II Stage	78 (22.22%)	56 (48–64)		
III Stage	209 (59.54%)	54 (46–60)		
IV Stage	27 (7.69%)	54 (46–60)		
Tumor resection method (*n*, %)				
Ultra-low anterior resection	148 (42.17%)	56 (50–61.75)	H = 54.903	<0.001
Low anterior resection	94 (26.78%)	56 (50–61.75)		
High anterior resection	51 (14.53%)	43 (40–53)		
Transanal partial resection	58 (16.52%)	62 (54–72)		
Defunctioning stoma (*n*, %)				
Yes	205 (58.40%)	53 (46–58.5)	U = 11550.5	<0.001
No	146 (41.60%)	57 (48–67)		
Neoadjuvant treatment (*n*, %)				
Yes	164 (46.72%)	56 (49–65)	U = 11866.5	<0.001
No	187 (53.28%)	52 (43–59)		
Adjuvant treatment (*n*, %)				
Yes	217 (61.82%)	54 (46–61)	U = 13,678	0.351
No	134 (38.18%)	55 (48–60.25)		
Postoperative complications (*n*, %)				
Yes	33 (9.40%)	55 (46–61)	U = 5171.5	0.892
No	318 (90.60%)	55 (45.5–59.5)		

**Table 2:** Spearman correlations among continuous study variables.

Variable	IQR	Age	Tumor distance from anal verge	Postoperative hospital stay	Perceived social support	Psychological resilience	Social isolation	LARS score
Age	61 (55–69)	1						
Tumor distance from anal verge	5 (3–8)	0.158^**^	1					
Postoperative hospital stay	8 (7–10)	0.056	−0.015	1				
Perceived social support	54 (50–59)	−0.410^***^	−0.077	−0.021	1			
Psychological resilience	55 (47–64)	−0.516^***^	−0.095	0.004	0.671^***^	1		
Social isolation	55 (46–61)	0.015	−0.503^***^	0.005	−0.204^***^	−0.225^***^	1	
LARS score	29 (22–34)	−0.176^**^	−0.702^***^	0.022	0.094	0.095	0.595^***^	1

All coefficients are Spearman’s rho. Blank cells indicate the diagonal (*r* = 1.00). ^**^p < 0.01; ^***^p < 0.001 (two-tailed).

### 3.2 Factors associated with social isolation

Significant differences in social isolation scores were observed according to tumor location (H = 86.676, *p* < 0.001), tumor resection method (H = 54.903, *p* < 0.001), defunctioning stoma status (U = 11,550.5, *p* < 0.001), and neoadjuvant treatment(U = 11,866.5, *p* < 0.001). Specifically, patients with different tumor locations showed significant variations in social isolation levels. Patients with ultra-low rectal position had the highest median social isolation score (59, IQR: 54–67). A clear gradient was observed, with lower tumor location being associated with higher levels of social isolation. Similarly, social isolation scores differed significantly across tumor resection methods. Patients undergoing transanal partial resection had the highest median social isolation score (62, IQR: 54–72), followed by those undergoing ultra-low anterior resection (56, IQR: 50–61.75). In addition, patients without defunctioning stoma had significantly higher social isolation scores (median: 57, IQR: 48–67) compared with those with defunctioning stoma (median: 53, IQR: 46–58.5). Patients who received neoadjuvant treatment also showed higher levels of social isolation (median: 56, IQR: 49–65) than those who did not receive neoadjuvant treatment (median: 52, IQR: 43–59).(see Table 1).

In the correlation analysis, bowel symptoms were moderately and positively correlated with social isolation (*r_s_* = 0.595, *p* < 0.001), indicating that greater bowel symptom burden was associated with higher levels of social isolation. However, bowel symptoms were not significantly correlated with perceived social support or psychological resilience (all *p* > 0.05). Tumor distance from the anal verge showed a moderate negative correlation with social isolation (*r_s_* = −0.503, *p* < 0.001), suggesting that patients with tumors located closer to the anal verge tended to report higher levels of social isolation. Perceived social support (*r_s_* = −0.204, *p* < 0.001) and psychological resilience (*r_s_* = −0.225, *p* < 0.001) were both significantly and negatively correlated with social isolation, indicating that greater psychosocial resources were associated with lower levels of social isolation. No significant correlations were observed between age and social isolation (*r_s_* = 0.015, *p* = 0.784) or between postoperative hospitalization duration and social isolation (*r_s_* = 0.005, *p* = 0.929) (see Table 2).

Regarding correlations among candidate predictors, the LARS score showed a strong negative correlation with tumor distance from the anal verge (*r_s_* = −0.702, *p* < 0.001), indicating that tumors located closer to the anal verge were associated with more severe postoperative bowel symptoms. Perceived social support was strongly and positively correlated with psychological resilience (*r_s_* = 0.671, *p* < 0.001). In addition, age showed moderate negative correlations with both perceived social support (*r_s_* = −0.410, *p* < 0.001) and psychological resilience (*r_s_* = −0.516, *p* < 0.001) (see Table 2).

Consequently, the covariates entered in the regression models included tumor location, tumor distance from the anal verge, tumor resection method, defunctioning stoma, and neoadjuvant treatment. Tumor location was considered as a categorical indicator of the anatomical level of the tumor within the rectum, whereas tumor distance from the anal verge provided a continuous and more precise measure of tumor height. Tumor resection method represented the actual surgical treatment approach and extent of rectal resection, thereby capturing treatment-related differences in postoperative functional recovery. Defunctioning stoma was considered an important perioperative treatment factor that may influence bowel-function recovery and the resumption of daily activities and social participation. Neoadjuvant therapy was included as a preoperative treatment factor that may be associated with subsequent bowel dysfunction and postoperative recovery.

### 3.3 Hierarchical regression analysis

To examine the moderating effects of perceived social support and psychological resilience on the association between postoperative bowel symptoms and social isolation, two hierarchical regression models were constructed, including the perceived social support model (Models 1–2 and Model 3a-4a) and psychological resilience model (Models 1–2 and Models 3b–4b). Prior to the regression analyses, all continuous variables were mean-centered to reduce multicollinearity between main effect and interaction terms, and Variance inflation factors (VIFs) were calculated separately for the perceived social support and psychological resilience moderation models. The VIF values ranged from 1.025 to 9.355 in both the perceived social support model and the psychological resilience model. The VIFs for all predictor variables were below 10, indicating an acceptable level of multicollinearity. The highest VIF was observed for the dummy variable representing ultra-low tumor location (VIF = 9.355), which was attributable to its strong correlation with the distance from the anal verge (*r* = −0.69) and its structural correspondence with the surgical approach. Notably, the VIFs of the primary predictors of interest in this study—including postoperative bowel symptoms (LARS score), social support/psychological resilience, and their interaction term—were low (1.025–2.176), indicating that multicollinearity was unlikely to materially affect the estimates of the core regression model. The specific VIF values for all predictor variables are presented in Supplementary Table S3. Diagnostic assessment of the final moderation models indicated no substantial violations of linearity, homoscedasticity, or residual normality. Although a small number of observations showed relatively large studentized residuals or leverage values, Cook’s distance values remained well below 1, indicating that no highly influential observations were identified (reporting of regression assumptions see Supplementary Figures S1, S2).

In Model 1, covariates that were significantly associated with social isolation in the univariate analyses, including tumor location, tumor distance from the anal verge, tumor resection method, defunctioning stoma, and neoadjuvant treatment were entered first. The model was statistically significant (*F* = 14.863, *p* < 0.001), explaining 28.2% of the variance in social isolation (R^2^ = 0.282; adjusted R^2^ = 0.263). Among the covariates, only tumor distance from the anal verge was independently associated with social isolation (B = −0.784, 95% CI [−1.258, −0.310], *p* = 0.001).

In Model 2, the centered LARS score was added to the model to examine the association between postoperative bowel symptoms and social isolation after adjustment for covariates. The addition of LARS significantly improved model performance (ΔR^2^ = 0.124, *p* < 0.001), with the total explained variance increasing to 40.6% (R^2^ = 0.406; adjusted R^2^ = 0.388). LARS was positively associated with social isolation (B = 0.506, 95% CI [0.388, 0.625], *β* = 0.518, *p* < 0.001), indicating that greater postoperative bowel symptom burden was associated with higher levels of social isolation.

To examine the independent contribution of perceived social support after controlling for LARS and covariates, Model 3a further included centered perceived social support. The model explained an additional 4.8% of the variance in social isolation (ΔR^2^ = 0.048, *p* < 0.001), with the total explained variance increasing to 45.3% (R^2^ = 0.453; adjusted R^2^ = 0.436). Perceived social support was negatively associated with social isolation (B = −0.364, 95% CI [−0.495, −0.232], *β* = −0.224, *p* < 0.001), suggesting that higher levels of perceived social support were associated with lower levels of social isolation. In Model 4a, the interaction term between bowel symptoms and perceived social support (LARS_centered × perceived social support_centered) was entered to test the moderating effect of perceived social support. The interaction term was significantly associated with social isolation (B = −0.020, 95% CI [−0.032, −0.009], *β* = −0.136, *p* = 0.001), and the explained variance further increased (ΔR^2^ = 0.018, *p* = 0.001). The final model explained 47.1% of the variance in social isolation (R^2^ = 0.471; adjusted R^2^ = 0.453). The significant negative coefficient of the interaction term indicated that perceived social support significantly moderated the association between postoperative bowel symptoms and social isolation. Specifically, higher levels of perceived social support weakened—but did not eliminate—the positive association between bowel symptom burden and social isolation, and the small increment in explained variance (ΔR^2^ = 0.018) suggested that this buffering effect was modest in magnitude. Detailed regression results are presented in Table 3.

**Table 3:** Hierarchical multiple regression analyses for the moderating effects of perceived social support and psychological resilience (*N* = 351).

Variables	B	SE B	*β*	95%CI	*t*	*p*-value
Model 1: Covariates						
Tumor location (reference: high position)						
Ultra-low position	4.353	3.136	0.188	[−1.816, 10.522]	1.388	0.166
Low position	4.163	2.611	0.164	[−0.973, 9.299]	1.594	0.112
Medium position	0.540	2.112	0.021	[−3.613, 4.694]	0.256	0.798
Tumor distance from anal verge	−0.784	0.241	−0.298	[−1.258, −0.310]	−3.252	0.001
Tumor resection method (reference: transanal partial resection)						
Ultra-low anterior resection	−3.029	1.692	−0.135	[−6.357, 0.299]	−1.790	0.074
Low anterior resection	−2.982	1.928	−0.119	[−6.773, 0.809]	−1.547	0.123
High anterior resection	−4.145	2.456	−0.132	[−8.977, 0.686]	−1.688	0.092
Defunctioning stoma	0.455	1.251	0.020	[−2.005, 2.914]	0.363	0.716
Neoadjuvant therapy	−0.933	1.109	−0.042	[−3.114, 1.248]	−0.842	0.400
*F*	14.863					<0.001
R^2^	0.282					
Adjusted R^2^	0.263					
Model 2: Main effect of LARS						
LARS_centered	0.506	0.060	0.518	[0.388, 0.625]	8.418	<0.001
*F*	23.204					<0.001
R^2^	0.406					
Adjusted R^2^	0.388					
ΔR^2^	0.124					<0.001
Model 3a: Main effect of social support						
Social support_centered	−0.364	0.067	−0.224	[−0.495, −0.232]	−5.436	<0.001
*F*	25.552					<0.001
R^2^	0.453					
Adjusted R^2^	0.436					
ΔR^2^	0.048					<0.001
Model 4a: Interaction term of LARS and perceived social support						
LARS_centered × Social support_centered	−0.020	0.006	−0.136	[−0.032, −0.009]	−3.408	<0.001
*F*	25.123					<0.001
R^2^	0.471					
Adjusted R^2^	0.453					
ΔR^2^	0.018					0.001
Model 3b: Main effect of psychological resilience						
Psychological resilience_centered	−0.259	0.040	−0.261	[−0.338, −0.181]	−6.497	<0.001
*F*	27.489					<0.001
R^2^	0.471					
Adjusted R^2^	0.454					
ΔR^2^	0.066					<0.001
Model 4b: Interaction term of LARS and psychological resilience						
LARS_centered × psychological resilience_centered	−0.011	0.004	−0.117	[−0.019, −0.004]	−2.963	0.003
*F*	26.508					<0.001
R^2^	0.485					
Adjusted R^2^	0.467					
ΔR^2^	0.013					0.003

Hierarchical multiple regression analyses for the moderating effects of perceived social support (Models 3a–4a) and psychological resilience (Models 3b–4b) on the association between postoperative bowel symptoms and social isolation. All continuous variables were mean-centered prior to analysis. B, unstandardized regression coefficient; SE B, standard error of the unstandardized coefficient; *β*, standardized regression coefficient; CI, confidence interval; *t*, *t*-test statistic.

Similarly, to examine the moderating role of psychological resilience, Model 3b included centered psychological resilience. The model showed a significant increase in explained variance (ΔR^2^ = 0.066, *p* < 0.001), with the total explained variance increasing to 47.1% (R^2^ = 0.471; adjusted R^2^ = 0.454). Psychological resilience was negatively associated with social isolation (B = −0.259, 95% CI [−0.338, −0.181], *β* = −0.261, *p* < 0.001), indicating that higher psychological resilience was associated with lower levels of social isolation. In Model 4b, the interaction term between LARS and psychological resilience (LARS_centered × psychological resilience_centered) was added. The interaction term was significantly associated with social isolation (B = −0.011, 95% CI [−0.019, −0.004], *β* = −0.117, *p* = 0.003), resulting in an additional 1.3% increase in explained variance (ΔR^2^ = 0.013, *p* = 0.003). The final model explained 48.5% of the variance in social isolation (R^2^ = 0.485; adjusted R^2^ = 0.467). These findings demonstrated that psychological resilience significantly moderated the association between postoperative bowel symptoms and social isolation. As in the social support model, the magnitude of the interaction effect was small (ΔR^2^ = 0.013), indicating a partial rather than substantial buffering effect. Detailed regression results are presented in Table 3.

### 3.4 Simple slope analysis and Johnson–Neyman analysis

To further elucidate the moderating effects of perceived social support and psychological resilience on the association between postoperative bowel symptoms and social isolation, simple slope analyses and Johnson–Neyman (J–N) analyses were conducted based on the significant interaction effects identified in the hierarchical regression models.

For perceived social support, simple slope analysis demonstrated that LARS scores were significantly and positively associated with social isolation at low, mean, and high levels of perceived social support (see Table 4; Figure 2). Specifically, when perceived social support was low (−1 SD, Social support_centered = −6.805), LARS scores were positively associated with social isolation (B = 0.645, SE = 0.069, *t* = 9.30, *p* < 0.001). This association remained significant at the mean level of perceived social support (Social support_centered = 0; B = 0.511, SE = 0.057, *t* = 8.97, *p* < 0.001). At high levels of perceived social support (+1 SD, Social support_centered = 6.805), the strength of the association between LARS scores and social isolation was further attenuated (B = 0.376, SE = 0.069, *t* = 5.31, *p* < 0.001). The decreasing slope estimates across increasing levels of perceived social support indicated that higher perceived social support weakened the strength of the association between postoperative bowel symptoms and social isolation; however, the association remained statistically significant even at high levels of perceived social support.

**Table 4:** Simple slope analysis of the moderating effects of perceived social support and psychological resilience.

Moderator	Simple slope (B)	SE	*t*	*p*	95% CI
Perceived social support					
Low (−1 SD)	0.645	0.069	9.30	<0.001	[0.510, 0.780]
Mean	0.511	0.057	8.97	<0.001	[0.399, 0.623]
High (+1 SD)	0.376	0.069	5.31	<0.001	[0.240, 0.512]
Psychological resilience					
Low (−1 SD)	0.640	0.072	8.86	<0.001	[0.499, 0.781]
Mean	0.519	0.056	9.23	<0.001	[0.409, 0.629]
High (+1 SD)	0.389	0.067	5.82	<0.001	[0.258, 0.521]

**Figure 2:**
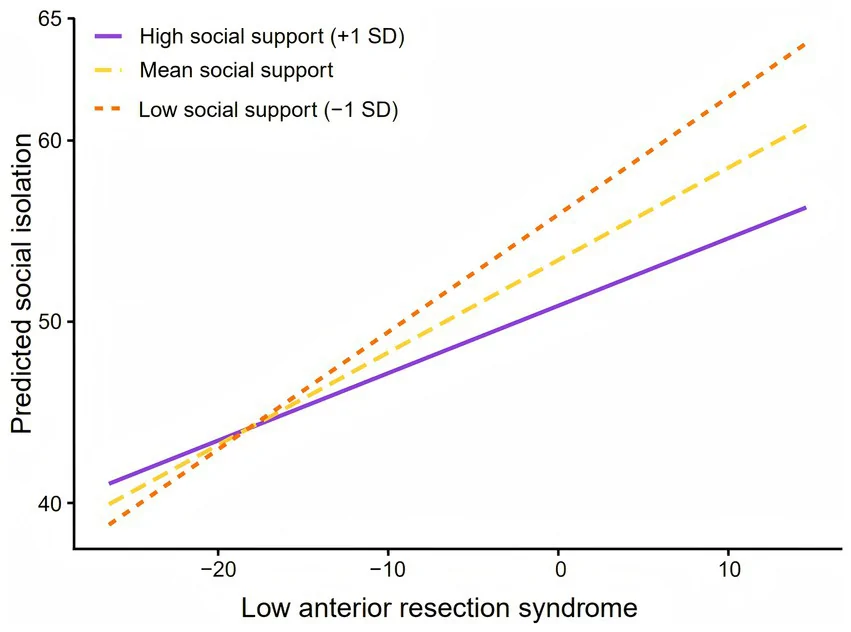
Simple slope analysis of social support.

The Johnson–Neyman analysis further identified the significant region of the moderating effect of perceived social support. The results showed that the association between LARS and social isolation was statistically significant when centered perceived social support was outside the interval of [14.89, 60.53]. Given that the observed range of centered social support in the current sample was −17.41 to 18.59, the upper boundary (60.53) was beyond the observed data range and therefore lacked practical interpretability. Within the observed range, the association between LARS and social isolation remained significant when centered perceived social support was below 14.89, whereas it became statistically nonsignificant only within the upper range of observed perceived social support (14.89 to 18.59). Because this nonsignificant region was located near the extreme end of the observed distribution, the identified value should be interpreted as a statistical region of significance rather than a clinically meaningful cutoff point (see Figure 3). These findings suggested that perceived social support may attenuate the association between postoperative bowel symptoms and social isolation. However, the nonsignificant region occurred only at the upper end of the observed social support distribution, indicating that complete buffering was not supported across most levels of perceived social support in this sample.

**Figure 3:**
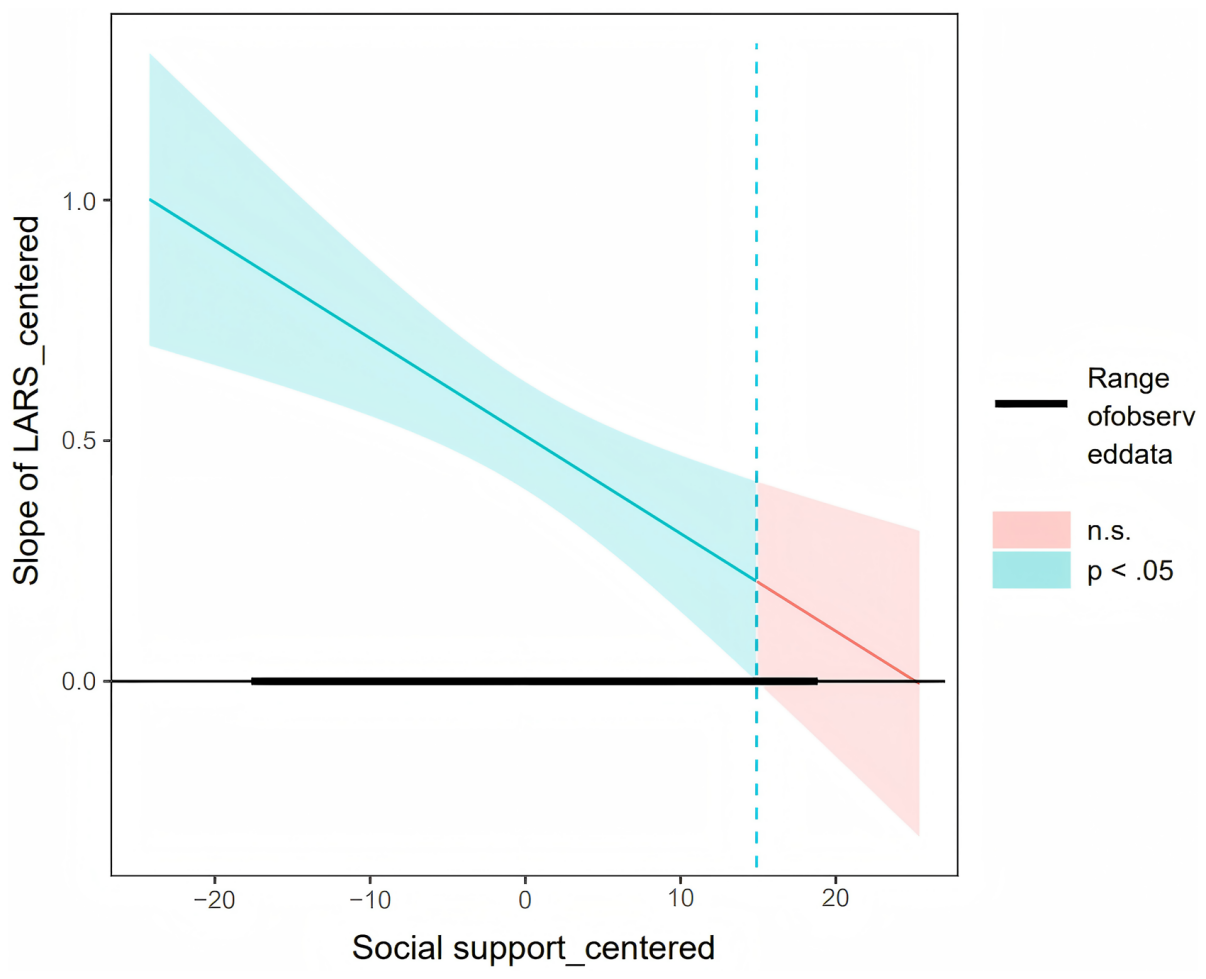
Johnson–Neyman analysis of social support.

For psychological resilience, simple slope analysis showed a similar moderating pattern. The association between LARS scores and social isolation was significant at low, mean, and high levels of psychological resilience (see Table 4; Figure 4). Specifically, at low psychological resilience (−1 SD, Psychological resilience_centered = −11.156), LARS scores were significantly associated with higher levels of social isolation (B = 0.640, SE = 0.072, *t* = 8.86, *p* < 0.001). At the mean level of psychological resilience (Psychological resilience_centered = 0), the association remained significant (B = 0.519, SE = 0.056, *t* = 9.23, *p* < 0.001). At high psychological resilience (+1 SD, Psychological resilience_centered = 11.156), the strength of the association between LARS scores and social isolation decreased (B = 0.389, SE = 0.067, *t* = 5.82, *p* < 0.001). These results indicated that higher psychological resilience attenuated the strength of the association between postoperative bowel symptoms and social isolation; however, the association remained statistically significant even at high levels of psychological resilience, suggesting a weakening rather than complete buffering effect.

**Figure 4:**
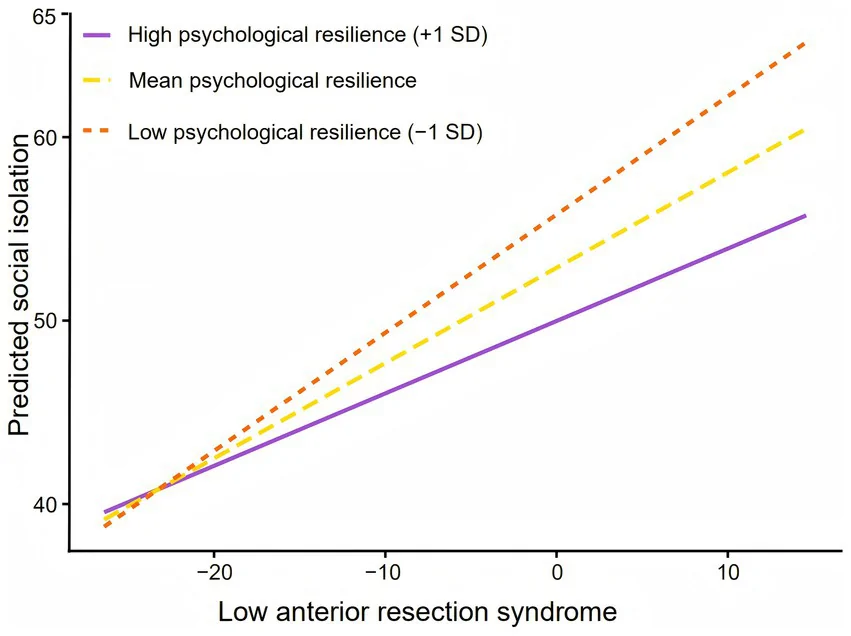
Simple slope analysis of psychological resilience.

The Johnson–Neyman analysis indicated that the effect of LARS on social isolation was significant when centered psychological resilience was outside the interval of [26.76, 137.74]. Considering that the observed range of centered psychological resilience was −26.85 to 26.15 in this study, both boundaries of the nonsignificant region fell outside the observed data range: the lower boundary (26.76) lay marginally above the observed maximum (26.15), and the upper boundary (137.74) was far beyond it. Consequently, the association between LARS and social isolation remained statistically significant across the entire observed range of psychological resilience, and no threshold of practical interpretability could be identified within this sample (see Figure 5). These findings suggested that psychological resilience may weaken the association between postoperative bowel symptoms and social isolation, but the observed levels of resilience were insufficient to reduce this association to a statistically nonsignificant level within the current sample.

**Figure 5:**
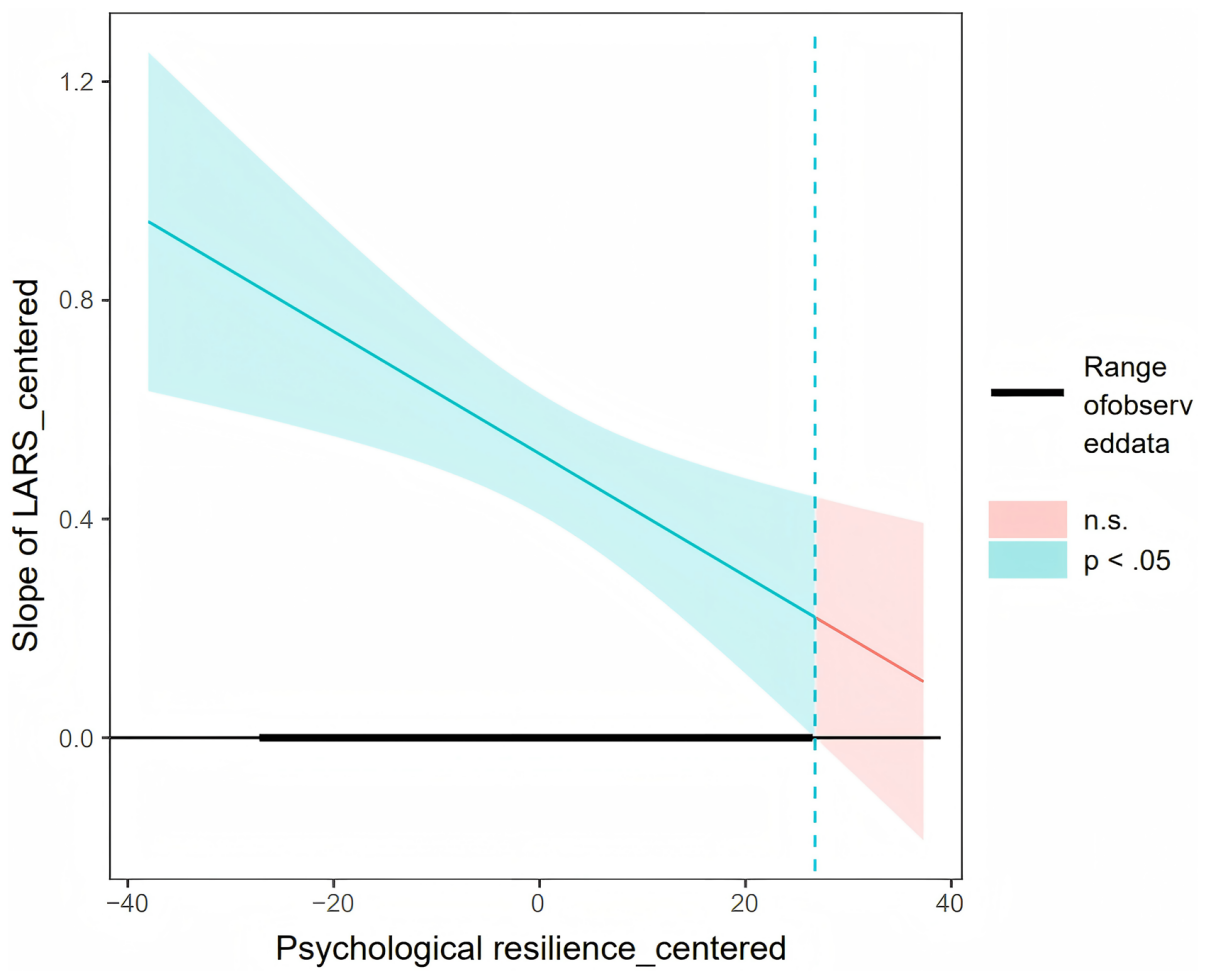
Johnson–Neyman analysis of psychological resilience.

### 3.5 Sensitivity analysis

To assess the robustness of the findings from the primary models, the two moderation models were re-estimated using three covariate specifications: S1, an unadjusted model with no covariate adjustment; S2, a parsimoniously adjusted model adjusting for distance from the anal verge, defunctioning stoma, and neoadjuvant therapy, while excluding tumor location because of its structural redundancy with distance from the anal verge; and S3, the fully adjusted primary model (Supplementary Table S4). Across the three specifications, the directions, statistical significance, and magnitudes of the main effects of postoperative bowel symptoms and the moderators, as well as their interaction effects, remained consistent. In the social support model, the main effect of bowel symptoms ranged from B = 0.506 to 0.623, while the interaction effect remained at B = −0.020 across all specifications. In the psychological resilience model, the main effect ranged from B = 0.506 to 0.624, and the interaction effect remained at B = −0.011 across all specifications. These findings indicate that the main and moderating effects remained stable across different model specifications.

## 4 Discussion

### 4.1 Main findings and significance of the study

Grounded in the stress-buffering theory, this study was the first to systematically examine the quantitative association between postoperative bowel symptoms and social isolation among patients with rectal cancer after sphincter-preserving surgery, while further investigating the moderating roles of perceived social support and psychological resilience in this association. The findings demonstrated that greater postoperative bowel symptom burden was significantly associated with higher levels of social isolation. Furthermore, both perceived social support and psychological resilience significantly attenuated the positive association between bowel symptoms and social isolation, although the magnitude of these moderating effects was relatively modest. This study extends previous research on the relationship of bowel symptoms with quality of life and psychological well-being by highlighting social isolation as an important psychosocial correlate after rectal cancer surgery. Moreover, the findings provide new insights into the potential moderating roles of external social resources and internal psychological resources in the association between postoperative bowel symptoms and social isolation. These results may contribute to a better understanding of psychosocial challenges related to impaired social functioning after sphincter-preserving surgery and may inform the development of integrated rehabilitation strategies combining symptom management and psychosocial support.

Beyond the psychosocial consequences observed at the individual level, these findings also highlight the importance of understanding postoperative social isolation from a broader public mental health perspective. Social functioning after rectal cancer surgery is not determined solely by symptom burden or individual psychological responses, but emerges through the interaction of individual, interpersonal, and structural factors. At the individual level, persistent bowel symptoms may contribute to symptom-related stigma, fear of negative evaluation, reduced confidence in social participation, and social withdrawal (36, 37). At the interpersonal level, support from family members, caregivers, and social networks may provide emotional reassurance, practical assistance, and encouragement, facilitating patients’ adaptation to symptom-related challenges and maintenance of social roles (38). At the structural level, social participation may be influenced by broader environmental conditions, including workplace demands, transportation accessibility, mobility limitations, availability of public toilets, and opportunities for social participation (39, 40). Therefore, postoperative social isolation should be recognized as a multidimensional public mental health concern shaped by the interaction between disease-related symptoms, social relationships, and the surrounding social environment.

### 4.2 Bowel symptoms and social isolation

In this study, postoperative bowel symptoms were moderately and positively correlated with social isolation among patients with rectal cancer undergoing sphincter-preserving surgery (*r_s_* = 0.595, *p* < 0.001). Furthermore, after adjusting for potential confounding factors, greater bowel symptom burden remained independently associated with higher levels of social isolation (B = 0.506, *β* = 0.518, *p* < 0.001). These findings provide quantitative evidence consistent with previous qualitative observations regarding the relationship between low anterior resection syndrome and psychosocial difficulties.

Consistent with our findings, a systematic review by Kim et al. (9), which synthesized evidence from 22 qualitative studies involving 415 patients, identified “living with uncertainty” and “experiencing social isolation and disconnection” as two major experiential themes among patients with LARS. Due to unpredictable bowel symptoms, such as urgency and fecal incontinence, patients often interrupt daily activities, restrict social participation, and experience uncertainty, social withdrawal, and psychological distress. Similarly, a qualitative focus group study by Ribas et al. (8) reported that patients with severe LARS frequently planned their activities around the availability of public toilets because of concerns about unpredictable bowel symptoms. Some patients even chose to remain at home to avoid potential embarrassment or uncontrollable bowel episodes, with a few reporting almost complete restriction of leaving home for up to two years, reflecting severe social withdrawal and isolation.

These findings suggest that although sphincter-preserving surgery can avoid permanent stoma formation and preserve intestinal continuity, persistent postoperative bowel symptoms may remain closely associated with difficulties in patients’ reintegration into normal social roles. Therefore, postoperative care should not focus solely on symptom management but should also consider the broader psychosocial difficulties associated with bowel symptoms. Clinicians should routinely monitor postoperative bowel symptoms and assess patients’ social participation, social withdrawal, and levels of social isolation.

### 4.3 The moderating role of perceived social support

The present study further demonstrated that perceived social support significantly moderated the association between bowel symptoms and social isolation. After controlling for relevant demographic and clinical factors, the interaction term between bowel symptoms and social support was significantly associated with social isolation (B = −0.020, 95% CI [−0.032, −0.009], *β* = −0.136, ΔR^2^ = 0.018, *p* = 0.001). However, the practical magnitude of this moderation effect should be interpreted cautiously. Although the negative interaction coefficient indicated that higher perceived social support was associated with a weaker relationship between bowel symptoms and social isolation, the interaction term accounted for only an additional 1.8% of the variance in social isolation (ΔR^2^ = 0.018), suggesting a modest moderation effect. Therefore, perceived social support may partially attenuate the adverse association between bowel symptoms and social isolation.

This finding is consistent with previous studies regarding the moderating effects of psychosocial resources on disease-related negative outcomes. A longitudinal study by Chang et al. (19). among patients after colorectal cancer surgery found that gastrointestinal symptom burden was associated with reduced quality of life, whereas higher levels of benefit finding attenuated this negative effect. Similarly, Carpenter et al. (41). reported that social support buffered the association between physical symptoms and cancer-related traumatic stress among gynecological cancer survivors. Higher perceived social support is associated with better adaptation to illness by providing emotional support, informational resources, and practical assistance. Such support may help patients maintain social participation despite bowel-related symptoms and may partly explain why the association between bowel symptoms and social isolation was weaker among patients with higher levels of social support (42). During postoperative follow-up, nurses should also assess patients’ levels of social support and social participation. For patients with severe bowel symptoms and insufficient social support, interventions such as strengthening family support, providing patient education, and facilitating peer support may help enhance social resources and promote recovery of social functioning.

It is noteworthy that the Johnson–Neyman analysis further clarified the range within which perceived social support significantly altered the association between bowel symptoms and social isolation. When centered perceived social support scores reached approximately 14.89 (corresponding to an actual score of 69.30) or higher, the association between bowel symptoms and social isolation became statistically nonsignificant within the observed range. However, this region represented only a relatively narrow range at the upper end of social support distribution rather than a clinical threshold indicating complete protection. Therefore, although higher perceived social support may substantially weaken the association between bowel symptoms and social isolation among individuals with very high levels of support, it should be considered as a partial protective resource rather than a factor that completely eliminates the psychosocial consequences of bowel symptoms.

### 4.4 The moderating role of psychological resilience

The present study further demonstrated that psychological resilience significantly moderated the association between postoperative bowel symptoms and social isolation. After controlling for relevant demographic and clinical factors, the interaction between bowel symptoms and psychological resilience was statistically significant (B = −0.011, *β* = −0.117, ΔR^2^ = 0.013, *p* = 0.003). Nevertheless, given that the interaction accounted for only 1.3% of the additional variance (ΔR^2^ = 0.013), the moderating effect of psychological resilience, although statistically robust, was limited in practical magnitude. The simple slope analysis further revealed that the strength of the association between bowel symptoms and social isolation decreased across increasing levels of psychological resilience (low resilience: B = 0.640; mean level: B = 0.519; high resilience: B = 0.389).

These findings are consistent with previous studies highlighting the protective role of psychological resilience in cancer populations. In a prospective study of breast cancer survivors, Manigault et al. (43) found that psychosocial resilience significantly buffered the association between cancer-related stress and depressive symptoms. Similarly, Li et al. (44), using hierarchical regression and simple slope analyses, demonstrated that psychological resilience moderated the relationship between emotional distress and post-traumatic growth among Chinese patients undergoing chemotherapy for breast cancer. Several possible explanations may account for this moderating pattern. Patients with higher levels of psychological resilience may be more likely to use adaptive coping strategies when facing unpredictable bowel symptoms, such as cognitively reframing symptom-related embarrassment, proactively planning toilet access, and flexibly modifying social activities rather than avoiding them altogether. Resilient individuals may also report better emotional regulation and a stronger perceived sense of control. These characteristics may be associated with greater maintenance of social participation and could partly explain the weaker association between bowel symptoms and social isolation observed among patients with higher resilience (45).

The Johnson–Neyman analysis further indicated that the association between bowel symptoms and social isolation remained statistically significant across the entire observed range of psychological resilience in the present sample. This pattern differs from the findings reported by Manigault et al. (43), in which the relationship between cancer-related stress and depressive symptoms became nonsignificant at high levels of resilience. Such discrepancies suggest that the buffering effect of psychological resilience may have limitations when the stressor involves persistent physiological dysfunction rather than transient psychological stress. Several explanations may account for this finding. First, unlike temporary psychological stressors, postoperative bowel symptoms following sphincter-preserving surgery represent a chronic and ongoing physical stressor accompanied by objective behavioral constraints, including dependence on toilet availability and the unpredictability of urgency and fecal incontinence. These functional limitations may directly restrict patients’ social participation regardless of their psychological coping capacity. Second, the present study assessed patients at one month after surgery, a period during which bowel symptoms are typically at their most severe and patients may not yet have developed effective symptom-management strategies (46). Under such circumstances, the protective effect of psychological resilience may be insufficient to fully offset the impact of severe bowel symptom burden.

Taken together, these findings suggest that psychological resilience can weaken the association between bowel symptoms and social isolation; however, within the observed range of resilience, it did not fully buffer this association. For patients experiencing severe bowel symptoms, interventions aimed solely at enhancing psychological resilience may be insufficient to fully restore social functioning. Comprehensive rehabilitation strategies, including bowel function training, dietary guidance, rehabilitation education, and the provision of family and social support, are therefore essential to mitigate the psychosocial consequences of postoperative bowel symptoms.

### 4.5 Clinical implications

The findings of this study have several important implications for clinical practice.

First, postoperative care should extend beyond the management of physical symptoms and incorporate assessment of psychosocial functioning into routine follow-up programs. While monitoring bowel symptom severity, healthcare providers should also identify patients at increased risk of impaired social functioning and provide timely psychological and social interventions.

Second, nurses should prioritize the assessment and enhancement of patients’ social support systems. Interventions such as caregiver education, strengthening family involvement, and facilitating peer support may help improve patients’ social adaptation and reduce the risk of social isolation.

Finally, clinical interventions should adopt a comprehensive approach integrating symptom management, psychological support, and social resource enhancement. Such multidimensional rehabilitation strategies may effectively facilitate patients’ reintegration into family, social, and daily life activities.

### 4.6 Limitations

This study provides quantitative evidence on the association between bowel symptoms and social isolation among patients with rectal cancer undergoing sphincter-preserving surgery, and further explores the moderating roles of perceived social support and psychological resilience. However, several limitations should be acknowledged. First, this was a single-center study conducted at a tertiary hospital in China, which may limit the representativeness and generalizability of the findings. In addition, the inclusion of participants who completed the 1-month follow-up may have introduced selection bias, and 57 of 408 participants were lost to follow-up (14.0%), which may have resulted in attrition bias. Second, social isolation, perceived social support, and psychological resilience were assessed using self-report measures and may therefore be subject to reporting bias. Third, although relevant covariates were adjusted for, residual confounding from unmeasured factors cannot be excluded. Finally, bowel symptoms and social isolation were assessed concurrently at the 1-month postoperative time point, which limits temporal and causal inference, and the use of only one postoperative assessment precluded evaluation of longitudinal changes.

## 5 Conclusion

This study demonstrated a significant positive association between postoperative bowel symptoms and social isolation among patients with rectal cancer following sphincter-preserving surgery. Furthermore, guided by the stress-buffering theory, our findings revealed that perceived social support and psychological resilience significantly moderated this association, although the magnitude of these moderating effects was modest. These findings highlight the importance of integrating bowel symptom management with psychosocial support to address postoperative social isolation and promote social functioning.

## Data Availability

The datasets presented in this article are not readily available due to patient confidentiality. Requests to access the datasets should be directed to the corresponding author.
