# Supplementary material for: Association between postoperative bowel symptoms and social isolation in patients with rectal cancer: the moderating effects of social support and psychological resilience
Source: Front Public Health. 2026 Oct 14;14:1963156. doi: 10.3389/fpubh.2026.1963156 (PMC13713419; doi:10.3389/fpubh.2026.1963156)
Supplement: Supplementary file 1 [file Supplementary_file_1.DOCX]

**Supplementary Materials**

**Table S 1. Comparison of baseline characteristics between Completed follow-up group and Lost to follow-up group**

| **Variable** | **Lost to follow-up (n=57)** | **Completed follow-up (n=351)** | **Statistics** | ***p*** |
| --- | --- | --- | --- | --- |
| **Gender (n, %)** |  |  |  |  |
| Male | 37 (64.91) | 214 (60.97) | χ² = 0.322 | 0.570 |
| Female | 20 (35.09) | 137 (39.03) |  |  |
| **Age (years) (IQR)** | 63 (58–72) | 61 (55–69) | Z = −1.567 | 0.117 |
| **BMI (n, %)** |  |  |  |  |
| Slender | 7 (12.28) | 21 (5.98) | χ² = 5.308 | 0.151 |
| Normal | 35 (61.40) | 195 (55.56) |  |  |
| Overweight | 12 (21.05) | 115 (32.76) |  |  |
| Obesity | 3 (5.26) | 20 (5.70) |  |  |
| **Level of education (n, %)** |  |  |  |  |
| Elementary school and below | 26 (45.61) | 118 (33.62) | χ² = 3.951 | 0.267 |
| Middle school | 20 (35.09) | 168 (47.86) |  |  |
| Junior college | 6 (10.53) | 40 (11.40) |  |  |
| Bachelor's degree and above | 5 (8.77) | 25 (7.12) |  |  |
| **Residence type (n, %)** |  |  |  |  |
| Rural | 24 (42.11) | 128 (36.47) | χ² = 0.667 | 0.414 |
| Towns | 33 (57.89) | 223 (63.53) |  |  |
| **Living status (n, %)** |  |  |  |  |
| Living alone | 8 (14.04) | 30 (8.55) | χ² = 5.045 | 0.080 |
| Living with spouse | 41 (71.93) | 295 (84.05) |  |  |
| Living with others | 8 (14.04) | 26 (7.41) |  |  |
| **Marital status (n, %)** |  |  |  |  |
| Married | 47 (82.46) | 304 (86.61) | χ² = 0.708 | 0.702 |
| Unmarried | 1 (1.75) | 5 (1.42) |  |  |
| Divorce or widowhood | 9 (15.79) | 42 (11.97) |  |  |
| **Working status (n, %)** |  |  |  |  |
| Employed | 24 (42.11) | 156 (44.44) | χ² = 0.110 | 0.947 |
| Resignation or retirement | 30 (52.63) | 177 (50.43) |  |  |
| Unemployed | 3 (5.26) | 18 (5.13) |  |  |
| **Primary family caregiver (n, %)** |  |  |  |  |
| No caregiver | 4 (7.02) | 25 (7.12) | χ² = 0.434 | 0.805 |
| Spouse | 33 (57.89) | 218 (62.11) |  |  |
| Other caregivers | 20 (35.09) | 108 (30.77) |  |  |
| **Tumor location (n, %)** |  |  |  |  |
| Ultra-low rectal position | 22 (40.00) | 124 (35.33) | χ² = 1.002 | 0.801 |
| Low rectal position | 15 (27.27) | 90 (25.64) |  |  |
| Medium rectal position | 12 (21.82) | 84 (23.93) |  |  |
| High rectal position | 6 (10.91) | 53 (15.10) |  |  |
| **Tumor Distance from the anal verge (cm) (IQR)** | 4 (3–6) | 5 (3–8) | Z = −1.264 | 0.206 |
| **TNM staging (n, %)** |  |  |  |  |
| Ⅰ Stage | 5 (10.00) | 36 (11.32) | χ² = 3.484 | 0.323 |
| Ⅱ Stage | 6 (12.00) | 70 (22.01) |  |  |
| Ⅲ Stage | 36 (72.00) | 188 (59.12) |  |  |
| Ⅳ Stage | 3 (6.00) | 24 (7.55) |  |  |
| **Tumor resection method (n, %)** |  |  |  |  |
| Ultra-low anterior resection | 30 (53.57) | 148 (42.17) | χ² = 8.178 | **0.042** |
| Low anterior resection | 19 (33.93) | 94 (26.78) |  |  |
| High anterior resection | 3 (5.36) | 51 (14.53) |  |  |
| Transanal partial resection | 4 (7.14) | 58 (16.52) |  |  |
| **Defunctioning stoma (n, %)** |  |  |  |  |
| Yes | 39 (69.64) | 205 (58.40) | χ² = 2.540 | 0.111 |
| No | 17 (30.36) | 146 (41.60) |  |  |
| **Neoadjuvant treatment (n, %)** |  |  |  |  |
| Yes | 24 (42.86) | 164 (46.72) | χ² = 0.290 | 0.590 |
| No | 32 (57.14) | 187 (53.28) |  |  |
| **Adjuvant treatment (n, %)** |  |  |  |  |
| Yes | 38 (66.67) | 213 (62.10) | χ² = 0.436 | 0.509 |
| No | 19 (33.33) | 130 (37.90) |  |  |
| **Postoperative complications (n, %)** |  |  |  |  |
| Yes | 8 (14.04) | 33 (9.40) | χ² = 1.165 | 0.281 |
| No | 49 (85.96) | 318 (90.60) |  |  |
| **Days in hospital after surgery(day) (IQR)** | 8 (7–10) | 8 (7–10) | Z = −0.447 | 0.655 |
| **Psychological resilience (IQR)** | 52 (45–60.5) | 55 (47–64) | Z = −0.816 | 0.415 |
| **Social support (IQR)** | 53 (48–58) | 54 (50–59) | Z = −0.539 | 0.590 |

**Note** : Comparisons between groups based on the raw data observed in practice. Values are n (%) for categorical variables and M (IQR) for continuous variables. Percentages are column percentages. χ² = Pearson chi-square test; Z = Mann–Whitney U test. Due to missing baseline data, the valid numbers for some variables were: TNM staging, n = 368 (lost to follow-up, n = 50; completed, n = 318); tumor location, n = 406 (55; 351); neoadjuvant treatment, tumor resection method, and temporary defunctioning stoma, n = 407 (56; 351); adjuvant treatment, n = 400 (57; 343); tumor distance from the anal verge, n = 401; postoperative hospital stay, n = 404.The apparent difference in tumor resection method (*p*= 0.042) should be interpreted with caution, as two cells had observed counts of fewer than five, which may compromise the reliability of the asymptotic chi-square approximation. Following a clinically justified regrouping of the surgical procedures into radical anterior resection and transanal partial resection, the between-group difference was no longer statistically significant (χ² = 3.292, *p* = 0.070).

**Table S 2. Number and proportion of missing data**

| Variable | Missing count | Missing proportion |
| --- | --- | --- |
| TNM staging | 33 | 9.4% |
| Tumor distance from anal verge | 3 | 0.9% |
| Adjuvant treatment | 8 | 2.3% |
| Postoperative hospital stay（day） | 1 | 0.3% |

**Table S 3. Variance inflation factors of predictors in moderation models**

| **Predictors** | **The VIF in Social support model** | **The VIF in Psychological resilience model** |
| --- | --- | --- |
| Tumor location |  |  |
| Ultra-low | 9.355 | 9.355 |
| Low | 5.535 | 5.535 |
| Medium | 3.267 | 3.267 |
| Tumor distance from the anal verge | 4.199 | 4.199 |
| Tumor resection method |  |  |
| Ultra-low anterior resection | 2.748 | 2.748 |
| Low anterior resection | 2.860 | 2.860 |
| High anterior resection | 2.937 | 2.937 |
| Defunctioning stoma | 1.481 | 1.481 |
| Neoadjuvant therapy | 1.208 | 1.208 |
| LARS_centered | 2.176 | 2.176 |
| Social support_centered | 1.037 | — |
| LARS_centered × Social support_centered | 1.025 | — |
| Psychological resilience_centered | — | 1.037 |
| LARS_centered × Psychological resilience_centered | — | 1.025 |

**Table S 4. Stability of main and interaction effects across alternative covariate sets (N = 351)**

| **A. Perceived social support moderation model** | | | |
| --- | --- | --- | --- |
| **Statistic** | **S1 Unadjusted** | **S2 Parsimonious** | **S3 Primary model** |
| B (LARS) | 0.623 (0.039) | 0.508 (0.054) | 0.506 (0.060) |
| β | 0.637 | 0.520 | 0.518 |
| *P* | <0.001 | <0.001 | <0.001 |
| B (social support ) | −0.356 (0.065) | −0.370 (0.065) | −0.364 (0.067) |
| β | −0.219 | −0.227 | −0.224 |
| *P* | <0.001 | <0.001 | <0.001 |
| B (LARS × social support) | −0.020 (0.006) | −0.020 (0.006) | −0.020 (0.006) |
| β | −0.137 | −0.137 | −0.136 |
| *P* | <0.001 | <0.001 | 0.001 |
| R² | 0.445 | 0.463 | 0.471 |
| ΔR² (interaction step) | — | 0.019 | 0.018 |
| **B. Psychological resilience moderation model** | | | |
| **Statistic** | **S1 Unadjusted** | **S2 Parsimonious** | **S3 Primary model** |
| B (LARS) | 0.624 (0.039) | 0.515 (0.054) | 0.506 (0.060) |
| β | 0.638 | 0.527 | 0.518 |
| *P* | <0.001 | <0.001 | <0.001 |
| B (psychological resilience) | −0.250 (0.039) | −0.257 (0.039) | −0.259 (0.040) |
| β | −0.252 | −0.259 | −0.261 |
| P | <0.001 | <0.001 | <0.001 |
| B (LARS × psychological resilience) | −0.011 (0.004) | −0.011 (0.004) | −0.011 (0.004) |
| β | −0.119 | −0.112 | −0.117 |
| P | 0.003 | 0.005 | 0.003 |
| R² | 0.457 | 0.475 | 0.485 |
| ΔR² (interaction step) | — | 0.012 | 0.013 |

**Note :** S1, an unadjusted model with no covariate adjustment; S2, a parsimoniously adjusted model adjusting for distance from the anal verge, defunctioning stoma, and neoadjuvant therapy, while excluding tumor location because of its structural redundancy with distance from the anal verge; and S3, the fully adjusted primary model. B, unstandardized regression coefficient, values in parentheses are standard errors; β, standardized coefficient;

**Table S 5. Hierarchical multiple regression analyses based on complete cases (N = 310)**

| **Variables** | **B** | **SE B** | **β** | **95% CI** | **t** | **P-value** |
| --- | --- | --- | --- | --- | --- | --- |
| **Model 1: Covariates** | | | | | | |
| Tumor location (ref: high position) |  |  |  |  |  |  |
| Ultra-low position | 4.821 | 3.533 | 0.203 | [−2.131, 11.773] | 1.365 | 0.173 |
| Low position | 4.844 | 2.851 | 0.189 | [−0.767, 10.454] | 1.699 | 0.090 |
| Medium position | 0.595 | 2.260 | 0.023 | [−3.853, 5.043] | 0.263 | 0.793 |
| Tumor distance from anal verge | −0.746 | 0.283 | −0.280 | [−1.302, −0.189] | −2.637 | 0.009 |
| Tumor resection method (ref: transanal partial resection) |  |  |  |  |  |  |
| Ultra-low anterior resection | −3.582 | 1.918 | −0.159 | [−7.356, 0.192] | −1.868 | 0.063 |
| Low anterior resection | −3.643 | 2.127 | −0.148 | [−7.829, 0.543] | −1.713 | 0.088 |
| High anterior resection | −5.782 | 2.672 | −0.184 | [−11.040, −0.524] | −2.164 | 0.031 |
| Defunctioning stoma | −0.470 | 1.328 | −0.021 | [−3.083, 2.144] | −0.354 | 0.724 |
| Neoadjuvant therapy | 1.159 | 1.164 | 0.052 | [−1.131, 3.449] | 0.996 | 0.320 |
| F | 14.932 |  |  |  |  | <0.001 |
| R² | 0.309 |  |  |  |  |  |
| Adjusted R² | 0.289 |  |  |  |  |  |
| **Model 2: Main effect of LARS** | | | | | | |
| LARS_c | 0.514 | 0.064 | 0.528 | [0.389, 0.639] | 8.083 | <0.001 |
| F | 22.855 |  |  |  |  | <0.001 |
| R² | 0.433 |  |  |  |  |  |
| Adjusted R² | 0.414 |  |  |  |  |  |
| ΔR² | 0.124 |  |  |  |  | <0.001 |
| **Model 3a: Main effect of social support** | | | | | | |
| Social support_c | −0.355 | 0.070 | −0.220 | [−0.493, −0.217] | −5.054 | <0.001 |
| F | 24.805 |  |  |  |  | <0.001 |
| R² | 0.478 |  |  |  |  |  |
| Adjusted R² | 0.459 |  |  |  |  |  |
| ΔR² | 0.045 |  |  |  |  | <0.001 |
| **Model 4a: Interaction (LARS × social support)** | | | | | | |
| LARS_c × SS_c | −0.018 | 0.006 | −0.122 | [−0.030, −0.006] | −2.942 | 0.004 |
| F | 24.043 |  |  |  |  | <0.001 |
| R² | 0.493 |  |  |  |  |  |
| Adjusted R² | 0.472 |  |  |  |  |  |
| ΔR² | 0.015 |  |  |  |  | 0.004 |
| **Model 3b: Main effect of psychological resilience** | | | | | | |
| Psychological resilience_c | −0.269 | 0.042 | −0.268 | [−0.352, −0.186] | −6.372 | <0.001 |
| F | 27.220 |  |  |  |  | <0.001 |
| R² | 0.501 |  |  |  |  |  |
| Adjusted R² | 0.483 |  |  |  |  |  |
| ΔR² | 0.068 |  |  |  |  | <0.001 |
| **Model 4b: Interaction (LARS × psychological resilience)** | | | | | | |
| LARS_c × PR_c | −0.011 | 0.004 | −0.115 | [−0.018, −0.003] | −2.803 | 0.005 |
| F | 26.180 |  |  |  |  | <0.001 |
| R² | 0.514 |  |  |  |  |  |
| Adjusted R² | 0.494 |  |  |  |  |  |
| ΔR² | 0.013 |  |  |  |  | 0.005 |

**Table S 6. Simple slope analysis based on complete-case (N = 310)**

| Moderator | Simple slope (B) | SE | t | P | 95% CI |
| --- | --- | --- | --- | --- | --- |
| **Perceived social support** | | | | | |
| Low (−1 SD) | 0.640 | 0.070 | 8.700 | <0.001 | [0.49, 0.78] |
| Mean | 0.510 | 0.060 | 8.470 | <0.001 | [0.39, 0.63] |
| High (+1 SD) | 0.390 | 0.070 | 5.200 | <0.001 | [0.24, 0.53] |
| **Psychological resilience** | | | | | |
| Low (−1 SD) | 0.650 | 0.080 | 8.530 | <0.001 | [0.50, 0.80] |
| Mean | 0.530 | 0.060 | 8.950 | <0.001 | [0.41, 0.65] |
| High (+1 SD) | 0.410 | 0.070 | 5.840 | <0.001 | [0.27, 0.55] |

**Table S 7. Johnson–Neyman analysis based on complete-case (N = 310)**

| Moderator | J–N  nonsignificant interval | Observed range | Boundary within observed range |
| --- | --- | --- | --- |
| Perceived social support | [15.95, 87.56] | [−17.42, 18.58] | 15.95 |
| Psychological resilience | [27.74, 162.69] | [−26.58, 26.42] | None |

S1(a) Histogram of regression standardized residuals

S1(b) normal P–P plot of regression standardized residuals

S1(c) scatterplot of standardized residuals versus standardized predicted values

**Supplementary Fig S1**. Diagnostic plots for the final perceived social support moderation model. (a) Histogram of regression standardized residuals; (b) normal P–P plot of regression standardized residuals; and (c) scatterplot of standardized residuals versus standardized predicted values. These plots were used to assess residual normality, linearity, and homoscedasticity.

S2(a) Histogram of regression standardized residuals

S2(b) normal P–P plot of regression standardized residuals

S2(c) scatterplot of standardized residuals versus standardized predicted values

**Supplementary Fig S2**. Diagnostic plots for the final psychological resilience moderation model. (a) Histogram of regression standardized residuals; (b) normal P–P plot of regression standardized residuals; and (c) scatterplot of standardized residuals versus standardized predicted values. These plots were used to assess residual normality, linearity, and homoscedasticity.
